# Combining Predictive Models of Mortality and Time-to-Discharge for Improved Outcome Assessment in Intensive Care Units

**DOI:** 10.3390/jcm14134515

**Published:** 2025-06-25

**Authors:** Àlex Pardo, Josep Gómez, Julen Berrueta, Alejandro García, Sara Manrique, Alejandro Rodríguez, María Bodí

**Affiliations:** 1 Department of Medicine and Surgery, Rovira Virgili University, 43201 Tarragona, Spain; josep.goal@gmail.com (J.G.); juberrueta.hj23.ics@gencat.cat (J.B.); smanriquemoreno@gmail.com (S.M.); ahr1161@yahoo.es (A.R.); mbodi.hj23.ics@gencat.cat (M.B.); 2 Critical Care Department, Joan XXIII University Hospital, 43005 Tarragona, Spain; alejgarcia.hj23.ics@gencat.cat; 3 Pere Virgili Health Research Institute, Joan XXIII University Hospital, Rovira Virgili University, 43005 Tarragona, Spain

**Keywords:** intensive care, machine learning, mortality, prediction model, reproducibility, critical care management

## Abstract

**Background:** The Patient Outcome Assessment and Decision Support (PADS) model is a real-time framework designed to predict both mortality and the likelihood of discharge within 48 h in critically ill patients. By combining these predictions, PADS enables clinically meaningful stratification of patient trajectories, supporting bedside decision-making and the planning of critical care resources such as nursing allocation and surgical scheduling. **Methods:** PADS integrates routinely collected clinical data: SOFA variables, age, gender, admission type, and comorbidities. It consists of two Long Short-Term Memory (LSTM) neural networks—one predicting the probability of death and the other the probability of discharge within 48 h. The combination places each patient into one of four states: alive/discharged within 48 h, alive/not discharged, dead within 48 h, or dead later. The model was trained using MIMIC-IV data, emphasizing ease of implementation in units with electronic health records. Out of the 76,540 stays present in MIMIC-IV (53,150 patients), 32,875 (25,555 patients) were used after excluding those with short stays (<48 h) or life support treatment limitations. The code is open, well-documented, and designed for reproducibility and external validation. **Results:** The model achieved strong performance: AUCROC of 0.94 (±0.03) for mortality and 0.89 (±0.07) for discharge on training data, and 0.87 (±0.02) and 0.88 (±0.03), respectively, on the test set. As a comparison, benchmark models obtain worse accuracy (−13.4% for APS III, −19% for OASIS, and −7.4% for SAPS II). Predictions are visualized in an intuitive format to support clinical interpretation. **Conclusions:** PADS offers a transparent, reproducible, and practical tool that supports both individual patient care and the strategic organization of intensive care resources by anticipating short-term outcomes.

## 1. Background

During the last decade, several approaches, including machine learning models, have been tested to predict outcomes for adult patients admitted to intensive care units (ICU). In particular, mortality and length-of-stay (or time-to-discharge) are the most common topics of research.

Mortality prediction models in the state of the art usually rely on a large set of variables to improve performance. For instance, studies using the MIMIC-III dataset have considered 148, 138, or even 2000 features [1,2,3,4]. While this approach can enhance predictive accuracy, it often makes these models difficult to reproduce in other datasets. Some works [5,6,7,8] have used fewer features (less than 22), but their performance tends to be lower.

Research on length-of-stay or time-to-discharge prediction is not as extensive. Frequently, it is approached together with mortality prediction. For example, in [9], patient discharge within 24/48 h is predicted with moderate success (AUC 0.617) using a publicly available dataset. In [10], predictions are made using only 100 patients and 7 features, making generalization difficult. In [11], a method based on 360 features and Principal Component Analysis (PCA) is presented. While PCA improves performance, it complicates the interpretability of the model.

Some works have attempted to combine both mortality and discharge predictions. For example, ref. [11,12] achieved very good results using Random Forest, but in an internal cohort (approximately 12,000 ICU patients) with 91 variables. In [13], a high-performance model was trained for neonates using 26 features from the first 24 h in MIMIC-III. In [14], data from MIMIC-IV and eICU were used, but with a dataset larger than 100 features. In [15], a model with 27 features was developed using an internal cohort and validated externally, but no code was provided, and it is unclear how it would be presented to physicians in daily practice. In [16], a small dataset (956 patients) is used to predict ICU admission, mortality, and length-of-stay, resulting in low-performance models, probably due to the lack of data. Authors in [17] aim to predict mortality and length-of-stay with a minimal set of variables (12 in the baseline approach and 33 in the quantiles approach) using MIMIC-III; the results obtained were not promising and resulted in an AUC ROC of 0.78 in the case of the mortality predictor and 0.65 for the length-of-stay one.

Several studies highlight the potential of machine learning models to improve healthcare operations by optimizing patient flow, predicting admissions, and aiding in staff and bed management [18,19,20,21]. However, the lack of a transparent, reproducible, and scalable framework that presents results in a clinically friendly manner has limited their adoption in daily ICU practice. As a result, clinicians often rely on traditional scoring systems [22,23,24] that use only static data at ICU admission.

Despite significant advances in predictive modeling within the ICU setting, current approaches often remain impractical for bedside use. Many state-of-the-art models rely on extensive sets of variables—some incorporating up to 2000 features—which complicates reproducibility and hinders deployment across different ICU environments. Moreover, these models typically generate static predictions based on admission data alone, lacking flexibility to adapt to a patient’s evolving clinical trajectory. Consequently, their insights may have limited practical utility for real-time decision-making or operational tasks like staffing and resource allocation. Our work addresses this gap by proposing a streamlined, transparent framework that leverages a minimal set of routinely collected variables to provide dynamic, real-time risk stratification—offering clinically relevant patient-level insights that support timely decision-making and operational management.

The objective of our work is to develop an easy-to-implement, maintain, and scale framework to continuously monitor patients’ state during their stay in the ICU. This state is derived from the combination of mortality probability and the probability of being discharged in the next 48 h, using a minimal set of variables commonly collected in daily ICU practice.

## 2. Methods

### 2.1. Data Sources

We used Multi-parameter Intelligent Monitoring in Intensive Care (MIMIC-IV) [25], a freely available dataset, to develop PADS. The MIMIC-IV dataset was collected from the Beth Israel Deaconess Medical Center in Boston, MA, USA, containing approximately 60 k deidentified medical records from critically ill patients who were admitted to critical care units. The MIMIC-IV database includes physiologic data collected from bedside monitors, laboratory test results, medications, medical images, and clinical progress notes captured in the electronic health record from patients admitted to the ICU between 2008 and 2019. It contains patient demographics, vital signs, records of fluid and medication administration, results of laboratory tests, observations, and notes provided by care professionals [26]. The Institutional Review Board at the BIDMC granted a waiver of informed consent and approved the sharing of the research resource.

For this study, all MIMIC-IV patients were included except for those with stays shorter than 48 h (by definition, the proposed models need at least 48 h worth of data) and those with life support treatment limitation orders. Out of the 76,540 stays present in MIMIC-IV (53,150 patients), 32,875 stays (25,555 patients) were used after excluding those with short stays (<48 h) or life support treatment limitations.

The data extraction, processing, and models were developed in Python 3.11.11. All the code required to reproduce the results presented in this paper is shared in a public GitHub repository. However, data access to MIMIC-IV has to be requested to be able to fully reproduce the results.

### 2.2. Variables and Features

One of the main goals of this work is to implement a framework that is easily reproducible. Aiming for that, we have decided to use as variables the ones defining the SOFA score. It is a commonly used scoring system, computed from basic measurements that can be easily obtained from almost any EHR (Electronic Health Record). In addition to that, there are four additional variables considered important that have been included: gender, age, admission type, and comorbidities by means of the Charlson comorbidity index [27]. The targets of each model will be the ICU mortality and a variable for the discharge in the next 48 h, both of them binary.

In terms of data preprocessing, outliers have been excluded by clipping the data using the physiological range defined by the clinicians (see Table 1). Then, missing values have been imputed using the strategy also detailed in Table 1. Finally, data has been scaled using a min–max scaler based on training samples.

This imputation strategy reflects the hourly structure of the dataset, where missing values often indicate that certain measurements or interventions were not applicable at that time. For instance, zeros for drug infusion rates show that no infusion was ongoing. In other cases, the last known measurement was used as the best estimate of the patient’s current status. These rules were agreed upon with the clinical team to ensure they align with typical ICU workflows.

**Figure 1:**
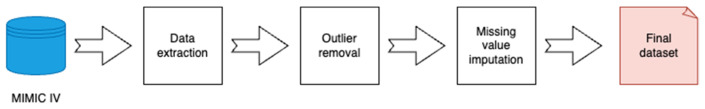
Data processing pipeline.

The data processing pipeline is detailed in Figure 1.

### 2.3. Libraries Used

Sharing the libraries used to develop this model is critical for reproducibility. For that reason, the Python version and the libraries are listed below:

▪ Python 3.11.11
▪ joblib == 1.3.2
▪ keras == 3.10.0
▪ matplotlib == 3.8.2
▪ numpy == 1.26.2
▪ pandas == 2.2.3
▪ scikit_learn == 1.4.2
▪ tensorflow == 2.18.0
▪ tqdm == 4.67.1

### 2.4. Model Development

**Figure 2:**
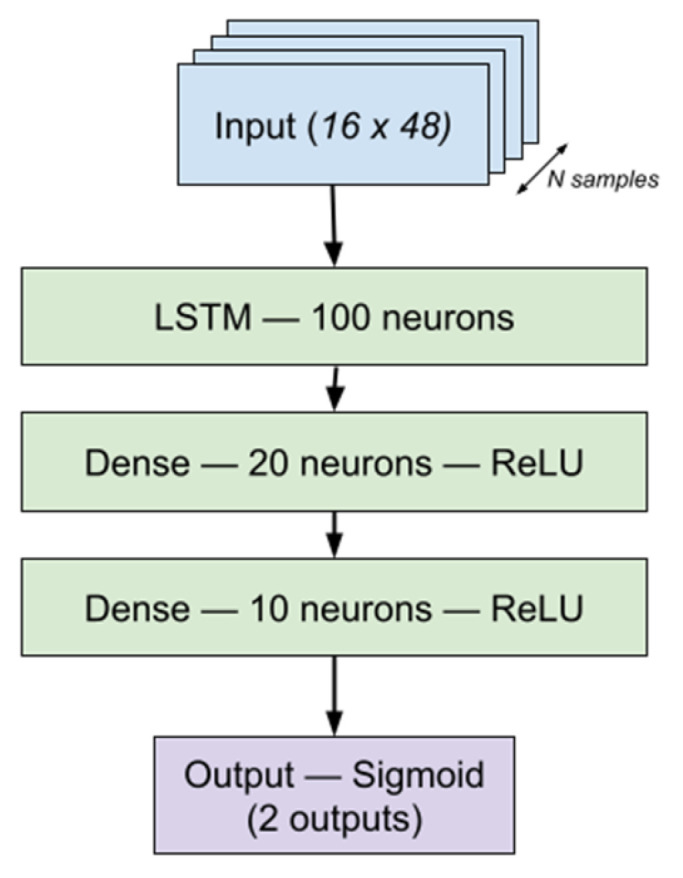
Neural network topology.

The architecture used in both neural networks is similar (see Figure 2): an LSTM [28] layer, two dense layers, and a binary output. For the LSTM layer, we have used 100 units and 20 and 10 neurons for the densely connected layers with ReLU activation and finally 2 outputs. All of these settings have been chosen using cross-validation. Models were trained using categorical cross-entropy loss with the Adam optimizer, incorporating both dropout layers and batch normalization to enhance generalization. Early stopping was implemented based on the validation set binary cross-entropy loss to prevent overfitting.

To address class imbalance, training samples were weighted inversely proportional to their class frequencies. Post-training, neural network outputs were calibrated using temperature scaling to improve probability calibration. Figure 2 shows the structure of the network.

For the training phase, not all the data has been used. In the case of the mortality model, only the last 48 h of data have been used to train the model in order to speed up the process and avoid overfitting on non-relevant data instances. We hypothesize that just providing the last 48 h will be enough for the model to be able to understand which features will decide if a patient lives or not. For the discharge prediction, we have considered three distinct 48 h segments within each patient’s ICU stay: the first 48 h, the 48 h in the middle of the stay, and the last 48 h. This strategy was chosen to capture variability across the different phases of a patient’s trajectory (early stabilization, evolving care, and pre-discharge status), while maintaining a balance between the two discharge prediction classes (<48 h and >48 h). This approach ensures that the model is trained on a diverse yet balanced dataset, minimizing the risk of bias due to overrepresentation of long stays. For the evaluation of the models, however, all the available data has been considered. In terms of the train and test split, we have chosen a 5-fold cross-validation.

The last part of the model is the fusion of both outcomes. It has been completed in a visual fashion in order to provide the clinician with a tool that has the outcomes of both models side-by-side. The following diagram describes the whole architecture of the proposed approach.

**Figure 3:**
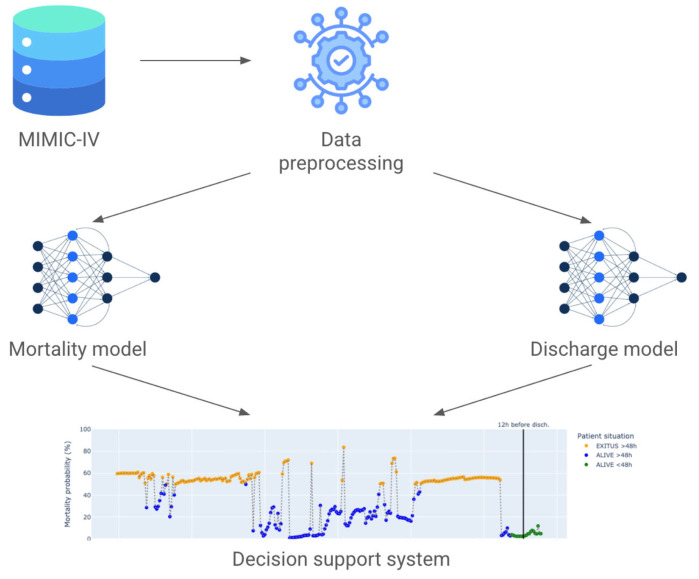
PADS flow diagram.

In Figure 3, the flowchart of the model is presented.

Model performance evaluation has been divided into three phases: train, test, and combined model. Next, all these phases are described.

In the training phase, categorical cross entropy has been used for the model training and AUC ROC for assessing the model performance on each fold.

In the test phase, AUC ROC has been used for assessing the model performance. The proposed approach for mortality prediction has also been tested against other methods (APACHE-III, OASIS, and SAPS-II).

In the case of the combined model, given that each outcome-prediction pair has different implications in a real-world scenario, we have designed a custom scoring matrix (see Table 2). This matrix assigns scores based on the “distance” between the predicted and actual states, which can be viewed as an ordered progression: *Alive <= 48 h*, *Alive > 48 h*, *Dead > 48 h*, and *Dead <= 48 h*. Errors are penalized more heavily the further apart the predicted and actual states are within this sequence. For example, misclassifying *Alive <= 48 h* as *Dead <= 48 h* represents the largest clinical discrepancy and receives the highest error score (3). In contrast, adjacent states—such as *Alive > 48 h* with *Alive <= 48 h* on one side and *Dead > 48 h* on the other side—are only assigned an error score of 1. This design ensures that critical mistakes are strongly penalized, while smaller misclassifications within clinically similar states are less severely weighted. This approach provides a more nuanced and clinically relevant assessment of model performance compared to standard metrics alone.

### 2.5. Model Training

The model was trained on a MacBook Pro with an M4 Pro processor and 24 GB of RAM. The dataset generation elapsed 38 min. In terms of model training, the mortality model training lasted 1 h 57 min, while the discharge model lasted 52 min.

### 2.6. Use of Generative AI Tools

During the manuscript preparation process, generative AI tools—Perplexity Sonar, Gemini 2.5 Flash, and GPT-4o—were used solely for minor text refinement, such as correcting grammar, improving sentence structure, and ensuring language clarity. These tools were not used for data analysis, result interpretation, or generating scientific content. All AI-generated suggestions were critically reviewed, edited, and approved by the authors to ensure accuracy and integrity.

## 3. Results

**Figure 4:**
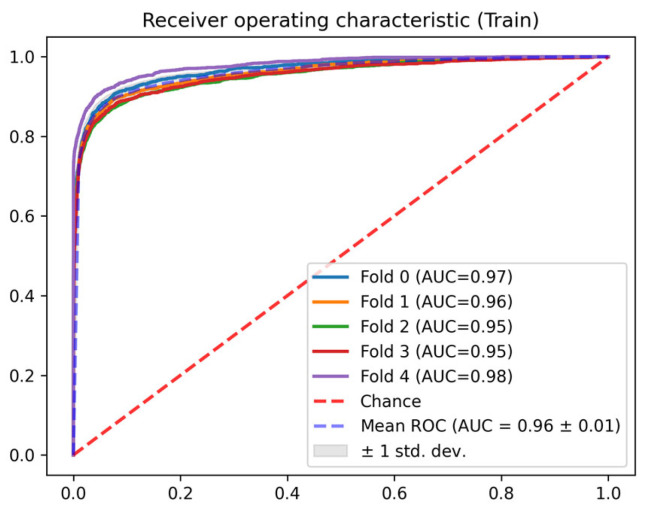
Train AUC ROC for the mortality model.

**Figure 5:**
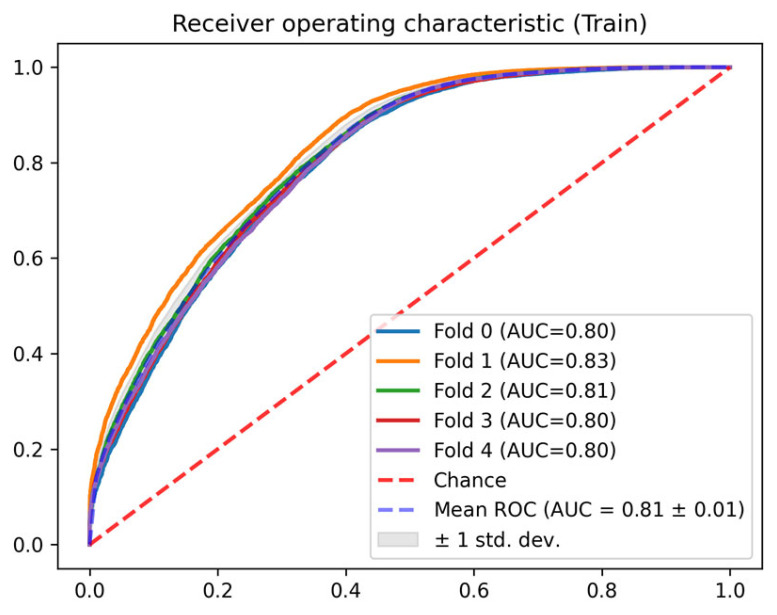
Train AUC ROC for the discharge prediction model.

In this section, the results of each of the phases described in Section 2.3 are detailed. In terms of the AUC ROC of the train sets, the mean AUC ROC is 0.96 ± 0.01 for the mortality prediction model (Figure 4) and 0.81 ± 0.01 for the discharge predictor (Figure 5), showing consistent results between the different folds. Similar results are observed for the test set. The in-admission models are evaluated at the admission, and the LSTM mortality model is evaluated using the last 48 h of every stay (the same data used for training).

**Figure 6:**
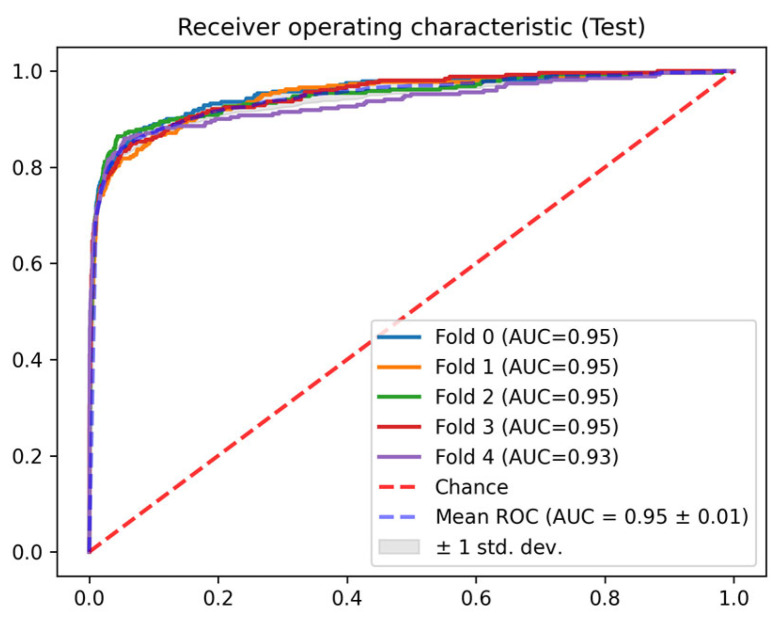
Test AUC ROC for the mortality model.

**Figure 7:**
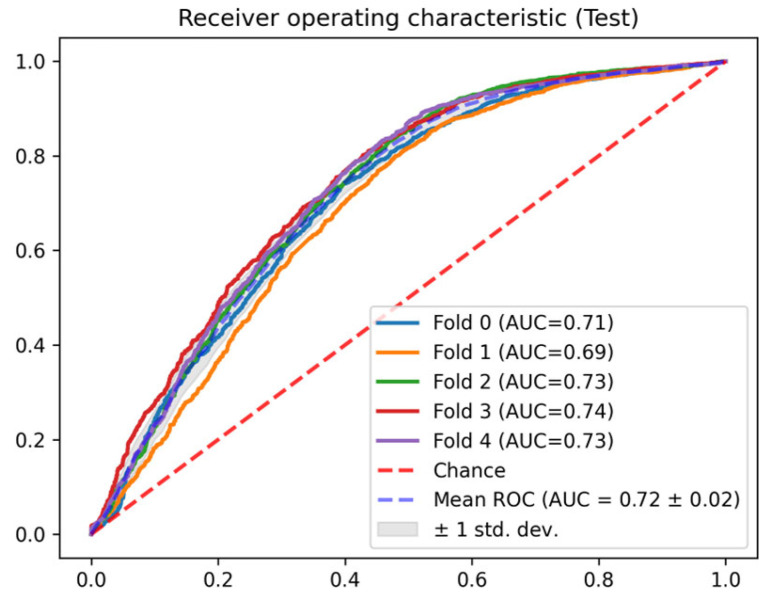
Test AUC ROC for the discharge prediction model.

Regarding the AUC ROC of the test sets, the mean AUC ROC is 0.95 ± 0.01 for the mortality prediction model (Figure 6) and 0.72 ± 0.02 for the discharge predictor (Figure 7), showing consistent results between the different folds. The in-admission models are evaluated at the admission, and the LSTM mortality model is evaluated using the last 48 h of every stay (the same data used for training).

In addition to the AUC ROC curves, precision, recall, f1-score, accuracy, and the Brier score have been analyzed (see Table 3 and Table 4). The results presented show robustness in the predictions, with relatively high f1-scores taking into account how unbalanced the dataset is. Also, the Brier score shows a good calibration of the model. Especially in the case of the discharge model, there is some deterioration in all the KPIs in the test set compared to the train set. This may indicate some degree of overfitting.

In addition to the AUC ROC, the accuracy of the mortality model is compared to state-of-the-art in-admission models (Table 5). The proposed mortality model presents greater accuracy for any length-of-stay bin.

After combining both models and applying the error scoring method proposed in Section 2.4, we observe that the average error per hour of stay is 0.585. As context, the best possible outcome would be 0, and, based on this dataset, the worst error value would be 2.66. The error matrix can be found in the Supplementary Materials.

**Figure 8:**
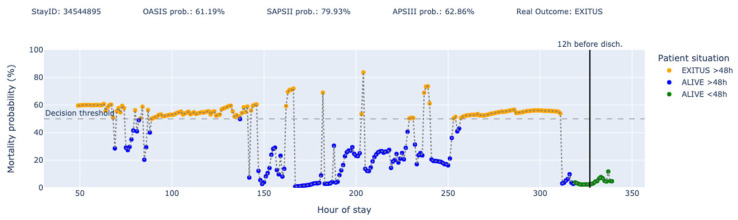
Predictions for stay ID 31369546.

Finally, we have designed the following plots (Figure 7 and Figure 8) as a proof of concept of what could be a decision support tool panel, where the y-axis represents the mortality probability and the colors represent each of the four states where a patient is situated at each moment. In addition, the actual outcome and the predictions made by in-admission models (OASIS, SAPS II, and APS III) are shown. The vertical black line indicates the point from which the patient will be discharged after 12 h.

As an example, in Figure 8, we see a patient for whom three of the most established scoring systems in the intensive care community (OASIS, SAPSII, and APSIII) predicted survival at the time of admission (all with mortality probabilities below 50%), but the patient ends up dying. Out of the 37 predictions generated by PADS, it only fails in 5 cases (with minimum scoring errors). At hours 62 and 64 (blue dots), the model predicts that the patient will survive beyond the next 48 h, while at hours 70, 71, and 72 (red dots), the model predicts the patient will die within the next 48 h.

**Figure 9:**
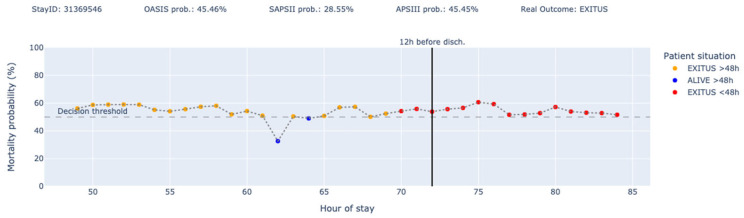
Predictions for stay ID 31359545.

On the other hand, in Figure 9, we have a case involving a very long stay, where the patient’s evolution is highly significant. We see how the three scoring systems (OASIS, SAPSII, and APSIII) predicted that the patient would die at the time of admission (all with mortality probabilities above 50%), but the patient ended up surviving after more than 14 days of hospitalization. In this case, all the blue and green points before the black line would be scoring the minimum error.

## 4. Discussion

In this study, we present a model capable of continuously framing the state of the patients according to the probability of short-term discharge and mortality during their stay in the ICU. The state is presented as a four-categorical output that combines the probability of death and the probability of being discharged in the next 48 h. This output is obtained hourly and allows clinicians to “situate” patients in relation to the distance of a certain outcome, enabling informed decision-making, which is valuable not only at the bedside but also for assisting in the organization of the critical care unit, such as predicting the unit’s occupancy rates, rescheduling non-critical surgical procedures, or staff allocation. The dynamic nature of this framework outperforms admission-based approaches, and the constrained set of variables makes it easy to implement in ICUs equipped with clinical information systems.

The accuracy of the presented model is lower than the top-performing state-of-the-art models [12,29]. However, the use of standard variables (SOFA, comorbidities, etc.) makes it easier to apply this model in any ICU with EHR. The top-performing models rely on extensive feature sets [1,2,3,12,14], making it much more difficult to implement at scale [30,31].

The dataset chosen for this work is the well-known MIMIC-IV, which is the most commonly used dataset in ICU prediction-related tasks. It contains a large number of stays (more than 70,000) and provides a high variety of validated variables with good time resolution, making it perfect for the proposed approach, as it requires a large number of cases for the neural network to train and an hourly resolution to account for sudden changes in patients’ medical conditions.

This approach not only considers the first 24 h of the stay (as classical models do) but also integrates information over the previous 48 h of the patient’s ICU stay, providing a more comprehensive assessment of the patient’s condition and trajectory. Moreover, longer stays will be equally represented, which is important in units with larger stays, such as those caring for patients with complex chronic conditions. Figure 8 reflects the importance of two key points in our project. On one hand, PADS is able to dynamically adjust the probabilities during the patient’s stay, rather than relying on a snapshot at a given moment. On the other hand, PADS is able to contextualize the probability of death with a temporal dimension indicating whether the death is imminent (<48 h) or not (>48 h). In the case of the stay depicted in Figure 8, our model also predicted death at the beginning, but beyond the next 48 h. This allows clinicians to classify the patient into a third severity level out of four possible and take actions accordingly.

The proposed method aims to become a management tool to support clinical decision-making and resource allocation in intensive care units using a straightforward and efficient machine learning framework that can be seamlessly integrated into existing clinical information systems. The usefulness in resource allocation can be extended to a regional scope if implemented in all the hospitals, being of special help in higher occupation periods.

The mortality prediction model presented in this study outperforms traditional scoring systems like APACHE-III, OASIS, and SAPS-II. This is evident from the higher accuracy of the model across all length-of-stay bins. The discharge prediction model also shows promising results, although it may benefit from further refinement to better capture the complexity of discharge decisions. The lower performance of the discharge prediction model can be attributed to the inherent complexity of discharge decisions, which are influenced not only by the patient’s clinical status but also by non-clinical factors such as bed availability, administrative policies, and social circumstances. To partially mitigate the impact of these factors, we excluded patients with life support treatment limitations, as such cases could systematically bias discharge outcome predictions. Despite these efforts, the multifactorial nature of discharge decisions likely explains the lower performance of the discharge prediction model compared to the mortality model. Nonetheless, in a real-time implementation for decision support, having a predictive label such as “alive < 48 h” or “alive > 48 h” can still help clinicians identify patients who might be closer to discharge, initiating relevant administrative or care planning tasks within their clinical context.

The proposed model has the potential to significantly enhance clinical decision-making by providing a continuous and dynamic assessment of patient outcomes. This can lead to more timely and effective interventions, reducing morbidity and mortality rates. The model’s ability to integrate information over the entire ICU stay will also help in better managing patients with complex conditions, ensuring that their care is tailored to their evolving needs.

Beyond the technical performance of the model, a key consideration for the practical utility of the PADS framework is its integration into clinical workflows. Implementing PADS would involve mapping the required input variables (SOFA score components, patient demographics, and comorbidities) to data sources in the local electronic health record (EHR) system and ensuring continuous, real-time data feeds. Potential barriers include variability in EHR data structures across hospitals, the need for rigorous data quality control, and ensuring that clinicians can easily interpret and trust the dynamic risk assessments produced by the model. To address these challenges, the framework has been designed with simplicity and interoperability in mind, using a minimal set of standardized variables and offering open-source, well-documented code to support local adaptation and validation. Close collaboration with clinical staff will also be crucial to ensure that model outputs are integrated in a way that supports decision-making without disrupting established care processes.

While the model demonstrates high predictive performance, it is not without limitations. For instance, the discharge of a patient is not always driven by the patient’s medical condition alone but can also be influenced by other factors such as the availability of beds. In the case of the discharge prediction model, in particular, it may benefit from further refinement to better capture the complexity of discharge decisions. Additionally, the MIMIC-IV dataset spans from 2008 to 2019, during which ICU practices, patient demographics, and care standards may have evolved. This temporal variability could potentially introduce data shifts, and model retraining or recalibration may be needed when applying our framework to more recent or locally specific data. The model’s performance on external datasets will also need to be evaluated to ensure its generalizability. Future work will focus on addressing these limitations and exploring the potential of the model in real-world clinical settings.

Furthermore, while the MIMIC-IV dataset provides a large and diverse cohort for model development, it may still contain inherent biases related to patient demographics, local ICU practices, and data collection protocols that could influence model performance. Additionally, real-time implementation of the PADS framework in different ICUs may encounter technical and logistical challenges, such as integrating dynamic predictions into existing clinical information systems and ensuring continuous data availability. From an ethical perspective, careful attention must be paid to data governance, patient privacy, and the potential for unintended consequences of automated predictions in critical care settings. Future work should focus on validating the framework in diverse clinical environments and exploring strategies to mitigate these challenges.

The architecture of Long Short-Term Memory (LSTM) networks naturally lends itself to transfer learning, making it an ideal choice for adaptable models [32,33]. This versatility allows the base LSTM model to be fine-tuned by modifying specific layers with local data, effectively tailoring the results to the target environment. Transfer learning has gained significant traction in recent years, demonstrating its effectiveness across medical domains [34]. Furthermore, this approach aligns well with federated learning principles, where model layers, rather than sensitive data, are shared between healthcare institutions [35,36]. This ensures that confidential information remains within each organization while still benefiting from collaborative model improvement, making it particularly valuable in privacy-sensitive sectors such as healthcare.

Beyond the ICU setting, the dynamic and modular nature of the PADS framework makes it well-suited for other high-dependency care environments, such as step-down units, post-surgical recovery areas, and general medical wards where continuous monitoring and resource planning are also critical. Its minimal input requirements and transparent architecture facilitate integration with existing clinical decision support systems, offering an opportunity to complement and enhance patient care pathways across different hospital departments. In future work, the framework could also be extended to incorporate other outcome predictions, such as risk of readmission or long-term functional outcomes. Furthermore, exploring federated learning approaches could enable institutions to collaboratively refine the model while ensuring data privacy and governance, supporting a more robust and generalizable clinical decision-making tool.

## 5. Conclusions

The presented approach combines a mortality model with a discharge predictor. The former is outperforming state-of-the-art admission-time-based methods, and the latter provides a layer of error correction on top of the mortality model. Both methods allow for continuous stratification of patients’ status and are presented as a decision support tool for clinicians using the visual representation proposed.

In terms of future work, our efforts will be focused on testing this approach on additional databases as well as testing it in an actual ICU. We will create and validate a real-time analysis tool with clinicians. Additionally, we plan to explore the use of more advanced machine learning techniques, such as multi-view learning and ensemble methods, to further improve the model’s performance. Also, explainability tools have been tested without success, but we plan to test additional approaches.

In conclusion, we successfully developed a model capable of situating patients in a risk state that contextualizes their probability of death with their probability of being discharged in the next 48 h. The output allows clinicians not just to monitor patients’ last state but also their path during their stay in the ICU. The presented framework has been designed for the model to be accessible, understandable, and usable for the scientific community, so we encourage researchers to test it using data from different ICUs.

## Figures and Tables

**Table 1:** Description of the variable units, missing value imputation method for each variable, and outlier removal and its result.

		Missing Value Imputation	Outlier Removal		Outlier Removal Result			
Variable	Unit	Method	Physiological Range		Mean		Std	
			Min	Max	Before	After	Before	After
Epinephrine rate	mcg/kg/min	Zero	0	10	0.060	0.060	0.148	0.148
Norepinephrine rate	mcg/kg/min	Zero	0	5	0.128	0.128	0.136	0.128
Dopamine rate	mcg/kg/min	Zero	0	50	6.335	6.140	15.125	4.342
Dobutamine rate	mcg/kg/min	Zero	0	40	4.550	4.550	2.529	2.529
Mean blood pressure (min. value)	mmHg	Last known value	10	150	78.744	78.707	15.711	15.441
PaO2/FiO2 Ratio (non-ventilated)	mmHg	Zero	50	600	248.921	247.347	113.810	105.835
PaO2/FiO2 Ratio (ventilated)	mmHg	Zero	50	600	248.711	244.400	134.976	114.530
Bilirubin (max. value)	mg/dL	Last known value	0.1	70	3.880	3.878	7.176	7.163
Creatinine (max. value)	mg/dL	Last known value	0.2	20	1.574	1.574	1.513	1.502
Platelets (min. value)	K/uL	Last known value	5.0	2000	202.572	202.570	134.076	134.047
Glasgow coma score	--	Last known value	--	--	14.32	1.71	14.32	1.71
Admission age	years	--	--	--	63.86	16.16	63.86	16.16
Admission type	--	--	--	--	--	--	--	--
Charlson comorbidity index	--	--	--	--	5.89	2.98	5.89	2.98

**Table 2:** Definition of the custom scoring model.

	Groundtruth				
		Alive <= 48 h	Alive > 48 h	Dead <= 48 h	Dead > 48 h
**Prediction**	Alive <= 48 h	0	1	2	3
	Alive > 48 h	1	0	1	2
	Dead <= 48 h	2	1	0	1
	Dead > 48 h	3	2	1	0

**Table 3:** Mortality model performance.

	Fold	AUC ROC	Precision	Recall	F1-Score	Accuracy	Brier Score
Train	0	0.968	0.765	0.987	0.784	0.972	0.039
	1	0.961	0.770	0.985	0.781	0.971	0.043
	2	0.953	0.730	0.988	0.772	0.971	0.049
	3	0.953	0.735	0.988	0.773	0.971	0.055
	4	0.979	0.763	0.998	0.850	0.982	0.029
Test	0	0.954	0.762	0.984	0.775	0.969	0.041
	1	0.949	0.739	0.984	0.754	0.968	0.046
	2	0.949	0.742	0.985	0.768	0.968	0.051
	3	0.951	0.694	0.990	0.754	0.972	0.055

**Table 4:** Discharge model performance.

	Fold	AUC ROC	Precision	Recall	F1-Score	Accuracy	Brier Score
Train	0	0.799	0.944	0.475	0.817	0.751	0.184
	1	0.833	0.943	0.531	0.828	0.771	0.172
	2	0.812	0.948	0.483	0.817	0.753	0.169
	3	0.804	0.937	0.499	0.815	0.753	0.173
	4	0.804	0.944	0.491	0.817	0.755	0.178
Test	0	0.710	0.894	0.401	0.772	0.691	0.227
	1	0.690	0.883	0.410	0.770	0.689	0.237
	2	0.726	0.902	0.449	0.788	0.715	0.209
	3	0.739	0.884	0.460	0.793	0.719	0.206

**Table 5:** Accuracy for in-admission models and LSTM mortality predictors.

LOS (h)	APS III	OASIS	SAPS II	LSTM 12 h Before Discharge
**(0, 24]**	63.48%	60.84%	77.13%	
**(24, 36]**	68.17%	65.54%	82.62%	
**(36, 48]**	64.92%	63.41%	76.04%	
**(48, 60]**	65.40%	60.42%	69.31%	75.94%
**(60, 72]**	64.49%	59.97%	69.02%	72.31%
**(72, 84]**	64.19%	60.01%	64.94%	70.07%
**(84, 96]**	66.46%	60.67%	64.59%	74.16%
**(96, 108]**	64.10%	58.28%	64.31%	78.46%
**(108, 120]**	65.39%	59.25%	65.08%	78.74%
**[120, inf)**	63.08%	59.65%	61.40%	75.53%

## Data Availability

The original data presented in the study is openly available in MIMIC-IV at https://doi.org/10.13026/kpb9-mt58.

## Supplementary Materials

The following supporting information can be downloaded at: https://www.mdpi.com/article/10.3390/jcm14134515/s1.

## References

[1] Johnson A.E.W., Mark R.G. (2017). Real-time mortality prediction in the Intensive Care Unit. AMIA Annu. Symp. Proc..

[2] Zhao S., Tang G., Liu P., Wang Q., Li G., Ding Z. (2023). Improving mortality risk prediction with routine clinical data: A practical machine learning model based on eICU patients. Int. J. Gen. Med..

[3] Majhi B., Kashyap A. (2023). Wavelet based ensemble models for early mortality prediction using imbalance ICU big data. Smart Health.

[4] Jiang J., Yu X., Wang B., Ma L., Guan Y. (2023). DECAF: An interpretable deep cascading framework for ICU mortality prediction. Artif. Intell. Med..

[5] Caicedo-Torres W., Gutierrez J. (2019). ISeeU: Visually interpretable deep learning for mortality prediction inside the ICU. J. Biomed. Inform..

[6] Liu M., Guo C., Guo S. (2023). An explainable knowledge distillation method with XGBoost for ICU mortality prediction. Comput. Biol. Med..

[7] Ishii E., Nawa N., Hashimoto S., Shigemitsu H., Fujiwara T. (2023). Development, validation, and feature extraction of a deep learning model predicting in-hospital mortality using Japan’s largest national ICU database: A validation framework for transparent clinical Artificial Intelligence (cAI) development. Anaesth. Crit. Care Pain Med..

[8] Thorsen-Meyer H.C., Nielsen A.B., Nielsen A.P., Kaas-Hansen B.S., Toft P., Schierbeck J., Strøm T., Chmura P.J., Heimann M., Dybdahl L. (2020). Dynamic and explainable machine learning prediction of mortality in patients in the intensive care unit: A retrospective study of high-frequency data in electronic patient records. Lancet Digit. Health.

[9] Lajevardi-Khosh A., Jalali A., Rajput K.S., Selvaraj N. (2021). Novel Dynamic Prediction of Daily Patient Discharge in Acute and Critical Care. Conf. Proc. IEEE Eng. Med. Biol. Soc..

[10] Hasan M.N., Hamdan S., Poudel S., Vargas J., Poudel K. (2023). Prediction of length-of-stay at intensive care unit (ICU) using machine learning based on MIMIC-III database. Proceedings of the 2023 IEEE Conference on Artificial Intelligence (CAI).

[11] Ma X., Si Y., Wang Z., Wang Y. (2020). Length of stay prediction for ICU patients using individualized single classification algorithm. Comput. Methods Programs Biomed..

[12] Iwase S., Nakada T.A., Shimada T., Oami T., Shimazui T., Takahashi N., Yamabe J., Yamao Y., Kawakami E. (2022). Prediction algorithm for ICU mortality and length of stay using machine learning. Sci. Rep..

[13] Juraev F., El-Sappagh S., Abdukhamidov E., Ali F., Abuhmed T. (2022). Multilayer dynamic ensemble model for intensive care unit mortality prediction of neonate patients. J. Biomed. Inform..

[14] Al-Dailami A., Kuang H., Wang J. (2022). Predicting length of stay in ICU and mortality with temporal dilated separable convolutionand context-aware feature fusion. Comput. Biol. Med..

[15] Lim L., Gim U., Cho K., Yoo D., Ryu H.G., Lee H.C. (2024). Real-time machine learning model to predict short-term mortality in critically ill patients: Development and international validation. Crit. Care.

[16] Saadatmand S., Salimifard K., Mohammadi R., Kuiper A., Marzban M., Farhadi A. (2023). Using machine learning in prediction of ICU admission, mortality, and length of stay in the early stage of admission of COVID-19 patients. Ann. Oper. Res..

[17] Alghatani K., Ammar N., Rezgui A., Shaban-Nejad A. (2021). Predicting Intensive Care Unit Length of Stay and Mortality Using Patient Vital Signs: Machine Learning Model Development and Validation. JMIR Med. Inform..

[18] Pianykh O.S., Guitron S., Parke D., Zhang C., Pandharipande P., Brink J., Rosenthal D. (2020). Improving healthcare operations management with machine learning. Nat. Mach. Intell..

[19] Wei J., Zhou J., Zhang Z., Yuan K., Gu Q., Luk A., Brent A.J., Clifton D.A., Walker A.S., Eyre D.W. (2024). Predicting individual patient and hospital-level discharge using machine learning. Commun. Med..

[20] Cho Y.S., Hong P.C. (2023). Applying machine learning to healthcare operations management: CNN-based model for malaria diagnosis. Healthcare.

[21] Han Y., Li Y., Li Y., Yang B., Cao L. (2023). Digital twinning for smart hospital operations: Framework and proof of concept. Technol. Soc..

[22] Zimmerman J.E., Kramer A.A., McNair D.S., Malila F.M. (2006). Acute Physiology and Chronic Health Evaluation (APACHE) IV: Hospital mortality assessment for today’s critically ill patients. Crit. Care Med..

[23] Le Gall J.R., Lemeshow S., Saulnier F. (1993). A new Simplified Acute Physiology Score (SAPS II) based on a European/North American multicenter study. JAMA.

[24] Johnson A.E.W., Kramer A.A., Clifford G.D. (2013). A new severity of illness scale using a subset of Acute Physiology And Chronic Health Evaluation data elements shows comparable predictive accuracy. Crit. Care Med..

[25] Johnson A.E.W., Bulgarelli L., Shen L., Gayles A., Shammout A., Horng S., Pollard T.J., Hao S., Moody B., Gow B. (2023). MIMIC-IV, a freely accessible electronic health record dataset. Sci. Data.

[26] Goldberger A.L., Amaral L.A., Glass L., Hausdorff J.M., Ivanov P.C., Mark R.G., Mietus J.E., Moody G.B., Peng C.K., Stanley H.E. (2000). PhysioBank, PhysioToolkit, and PhysioNet: Components of a new research resource for complex physiologic signals. Circulation.

[27] Charlson M.E., Pompei P., Ales K.L., MacKenzie C.R. (1987). A new method of classifying prognostic comorbidity in longitudinal studies: Development and validation. J. Chronic Dis..

[28] Hochreiter S., Schmidhuber J. (1997). Long short-term memory. Neural Comput..

[29] Zeng G., Zhuang J., Huang H., Tian M., Gao Y., Liu Y., Yu X. (2023). Use of deep learning for continuous prediction of mortality for all admissions in intensive care units. Tsinghua Sci. Technol..

[30] Matheny M.E., Goldsack J.C., Saria S., Shah N.H., Gerhart J., Cohen I.G., Price W.N., Patel B., Payne P.R.O., Embí P.J. (2025). Artificial Intelligence In Health And Health Care: Priorities For Action. Health Affairs.

[31] Wardi G., Owens R., Josef C., Malhotra A., Longhurst C., Nemati S. (2023). Bringing the Promise of Artificial Intelligence to Critical Care: What the Experience With Sepsis Analytics Can Teach Us. Crit. Care Med..

[32] Shickel B., Tighe P.J., Bihorac A., Rashidi P. (2018). Deep EHR: A survey of recent advances in deep learning techniques for electronic health record (EHR) analysis. IEEE J. Biomed. Health Inform..

[33] Rajkomar A., Oren E., Chen K., Dai A.M., Hajaj N., Hardt M., Liu P.J., Liu X., Marcus J., Sun M. (2018). Scalable and accurate deep learning with electronic health records. NPJ Digit. Med..

[34] Li S., Li X., Yu K., Miao D., Zhu M., Yan M., Ke Y., D’Agostino D., Ning Y., Wu Q. (2024). Bridging data gaps in healthcare: A scoping review of transfer learning in biomedical data analysis. arXiv.

[35] Rieke N., Hancox J., Li W., Milletarì F., Roth H.R., Albarqouni S., Bakas S., Galtier M.N., Landman B.A., Maier-Hein K. (2020). The future of digital health with federated learning. NPJ Digit. Med..

[36] Miotto R., Wang F., Wang S., Jiang X., Dudley J.T. (2018). Deep learning for healthcare: Review, opportunities and challenges. Brief. Bioinform..

