# Supplementary material for: Combining Predictive Models of Mortality and Time-to-Discharge for Improved Outcome Assessment in Intensive Care Units
Source: J Clin Med. 2025 Jun 25;14(13):4515. doi: 10.3390/jcm14134515 (PMC12249738; doi:10.3390/jcm14134515)
Supplement: Supplementary file 1 [file jcm-14-04515-s001.zip › jcm-3641720-supplementary.pdf]

Supplementary Material

The error matrix (Figure 1) represents how many samples lay in each of the buckets depending on the true value and the predicted one.

Error is computed multiplying each cell by the error represented by each color and dividing it by the total number of samples in the dataset. According to the definition, the minimum error is 0 and the maximum error is 2.387. The error achieved using PADS is 0.585.

In terms of percentage, 46.92% of the samples are in the diagonal (error = 0) and 47.67% are in the error = 1 area. That means errors 2 and 3 only represent 5.41%. These numbers are detailed in Figure 2.

		Groundruth			
		Alive <=48h	Alive >48h	Dead >48h	Dead <=48h
Prediction	Alive <=48h	699	2,149	683	1,012
	Alive >48h	917,406	1,362,867	133,574	46,266
	Dead >48h	118,947	400,797	103,761	37,958
	Dead <=48h	511	2,112	1,605	2,811

Error = 3
Error = 2
Error = 1
Error = 0

Figure S1. Error matrix. Each cell corresponds to the total number of samples.

		Groundruth			
		Alive <=48h	Alive >48h	Dead >48h	Dead <=48h
Prediction	Alive <=48h	0.02%	0.07%	0.02%	0.03%
	Alive >48h	29.28%	43.50%	4.26%	1.48%
	Dead >48h	3.80%	12.79%	3.31%	1.21%
	Dead <=48h	0.02%	0.07%	0.05%	0.09%

Figure S2. Error matrix. Each cell corresponds to the percentage of samples.
